# Up regulation of Bax and down regulation of Bcl2 during 3-NC mediated apoptosis in human cancer cells

**DOI:** 10.1186/s12935-015-0204-2

**Published:** 2015-05-28

**Authors:** Mohammad Hassan Naseri, Majid Mahdavi, Jamshid Davoodi, Saeed Hesami Tackallou, Mahdi Goudarzvand, Shima Hallaj Neishabouri

**Affiliations:** Alborz University of Medical Sciences, Karaj, Iran; Baqiyatallah University of Medical Sciences (BMSU), Tehran, Iran; Department of Biology, Faculty of Natural Science, University of Tabriz, Tabriz, Iran; Institute of Biochemistry and Biophysics, University of Tehran, Tehran, Iran; Department of Biology, Central Tehran Branch, Islamic Azad University, Tehran, Iran

**Keywords:** Apoptosis, 4-aryl-4H-chromenes, Bax, Bcl2, 3-NC

## Abstract

**Background:**

Recently, we have reported the induction of apoptosis by *2-amino-4-(3-nitrophenyl)-3-cyano-7-(dimethylamino)-4H-chromene* (3-NC) in HepG2, T47D and HCT116 cells with low nano molar IC50 values. In this study, anti-proliferative effects of modified 4-aryle-4H-chromenes derivatives; *2-amino-4-(3-bromophenyl)-3-cyano-7-(dimethylamino)-4H-chromene* (3-BC), *2-amino-4-(3-trifluoromethylphenyl)-3-cyano-7-(dimethylamino)-4H-chromene* (3-TFC) and *2-amino-4-(4,5-methylenedioxyphenyl)-3-cyano-7-(dimethylamino)-4H-chromene* (4, 5-MC) were investigated in three human cancer cell lines. Compared to 3-NC none of the compounds displayed better anti-proliferative effect, although 3-BC appeared somewhat similar. Therefore 3-NC was selected for further studies.

**Methods and results:**

Treatment of HepG2, T47D and HCT116 cells with this compound induced apoptosis as visualized by fluorescence microscopic study of Hoechst 33258 stained cells. Induction of apoptosis was quantified by Annexin V/PI staining using flow cytometry.

Western blot analysis also revealed that 3-NC down-regulated the expression of anti-apoptotic protein Bcl2 and up-regulated pro-apoptotic protein Bax, in all of the cell lines. Nonetheless, HepG2 cell line was the most responsive to 3-NC as Bax and Bcl2 showed the most dramatic up and down regulation.

**Conclusion:**

Our previous finding that 3-NC down regulates Inhibitor of Apoptosis Proteins (IAPs) and the present observation that Bax is upregulated and Bcl2 is down regulated upon 3-NC treatment, this chromene derivative has the potential to overcome chemotherapy resistance caused by up regulation of these proteins.

## Introduction

Apoptosis is the prevalent form of program cell death characterized by condensation of nuclear chromatin, loss of plasma membrane phospholipid asymmetry, enzymatic cleavage of the DNA into oligonucleosomal fragments, and segmentation of the cells into membrane-bound apoptotic bodies [1, 2]. The Bcl2 family proteins are key regulators of apoptosis cell death. These related proteins share at least one of four homologous regions termed Bcl homology (BH) domains (BH1 to BH4) which control Bcl2 protein interactions. BH domains contribute at multiple levels to the function of these proteins in cell death and survival. Currently about 25 members of this family have been identified that based on functional studies and the conservation of BH domains can be divided into three subgroups, pro-survival Bcl2-like subgroup such as Bcl2, Bcl-xL, Mcl-1, Bcl-w and A-1 which suppress cell death, and pro-apoptotic Bax-like subgroup such as Bax, Bak, Bok and Bik which promote cell death [3–5]. In living cell, Bcl2 and Bcl-xL bind to the BH3 domains of pro-apoptotic family members, sequestering them and thereby inhibiting their ability to promote cell death [6, 7]. The third subgroup contains pro-apoptotic BH3-only proteins, such as Bad, Bid, Bim, Noxa and Puma which can interact with either anti-apoptotic proteins or pro-apoptosis members [3, 8]. This subgroup can either inhibit the anti-apoptotic molecules or directly activate pro-apoptotic Bax or Bak to induce apoptosis [9]. Although it is not fully understood how Bcl2 family proteins regulate the apoptotic pathway, it has been demonstrated biological functions of this protein family is dependent on protein-protein interactions [10–12].

It has been found that Bcl2 play an important role in resistance of cancer cells to chemotherapy or radiation therapy. Bcl2 can promote the expansion of neoplastic cell by preventing normal cell turnover caused by physiological cell death mechanisms [13, 14]. High expression of Bcl2 in various human cancers mediates the resistance of cancers to a wide range of chemotherapeutic drugs and γ-irradiation which act by inducing apoptosis in tumor cells. Therefore, the blocking of Bcl2 can restore the apoptotic process in tumor cells. In this regard, inhibitor molecules of Bcl2 may represent a new class of therapeutic agents for cancer treatment [15].

**Fig. 1:**
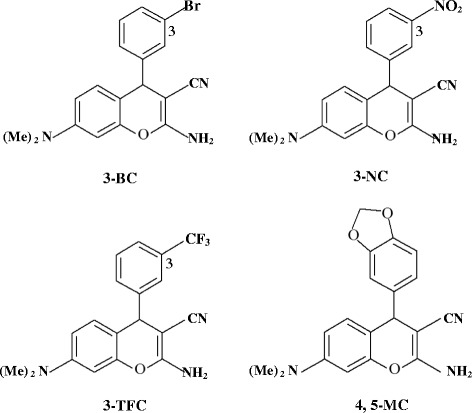
Chemical structure of the investigated 4-aryl-4H chromene derivatives

Despite considerable progress in identifying the causes of cancer and discovering new treatment strategies, significant number of cancers remains resistant to chemotherapy. Consequently, numerous efforts are under way to identify new compounds with anti-cancer activity the 4-aryl-4-H chromenes has been recently reported as a new group of potent anti-cancer compounds. These compounds bind to colchicine binding site in β tubulin and destabilize microtubular assembly resulting in cell cycle arrest at G2/M phase and apoptotic cell death [16–18]. They were also active in the multidrug-resistant cancer cell lines and showed promising antitumor activity in several in vivo mouse tumor models with evidence of antivascular activity [19, 20]. Further investigations showed that substituent position on the phenyl ring are more important, especially at the 3-position. Replacement of the electron withdrawing groups at this position of the phenyl ring was accompanied by a dramatic increase in cytotoxic effects of these compounds. We thus decided to position the strong electron withdrawing groups of nitro (-NO_2_), Trifluoromethyl (-CF_3_) and Bromo (-Br) on carbon 3 of the phenyl ring and also Methylenedioxy (-MDO) on carbons four and five of the ring (Fig. 1) to study their cytotoxic effects. In the previous study we reported concomitant activation of caspase-nine and down-regulation of IAP proteins as a mechanism of apoptotic death in HepG2, T47D and HCT-116 cells upon exposure to *2-amino-4-(3-nitrophenyl)-3-cyano-7-(dimethylamino)-4H-chromene* (3-NC) from 4-aryl-4-H chromenes family [21]. In the present study we demonstrate cytotoxic effects of the modified compounds of 4-aryl-4-H chromenes family on these cancer cell lines and show that 3-NC, in addition to down regulation of inhibitor of apoptosis proteins (IAPs), functions through down regulation of Bcl2 and up regulation of Bax.

## Materials and methods

### Materials

The cell culture medium (RPMI 1640), fetal bovine serum (FBS) and penicillin–streptomycin were purchased from Gibco BRL (life technolologies, Paisley, Scotland). The culture plates were obtained from Nunc (Denmark). Hoechst 33285 was purchased from Sigma Chemical Company (Germany). MTT assay kit was purchased from Roche (Germany). All antibodies, except β actin, including anti-Bcl2 and anti-Bax were purchased from Sigma (St Louis, MO, USA). β actin antibody was purchased from Alexis Biochemicals (San Diego, CA). Annexin-V-FITC kit was purchased from IQ product (Groningen, The Netherlands). All cell lines were obtained from Pasteur Institute of Iran (Tehran).

### General procedure for the preparation of 4-aryl-4H-chromene compounds

The 4-aryl-4H-chromene compounds were prepared according to the previously described method [16, 22]. Briefly, condensation of 3-dimethylaminophenol, a substituted benzaldehyde and malonitrile in ethanol in the presence of piperidine yielded the 4-aryl-4H-chromenes compounds. The structures of target compounds including *2-amino-4-(3-nitrophenyl)-3-cyano-7-(dimethylamino)-4H-chromene* (3-NC), *2-amino-4-(3-bromophenyl)-3-cyano-7-(dimethylamino)-4H-chromene* (3-BC), *2-amino-4-(3-trifluoromethylphenyl)-3-cyano-7-(dimethylamino)-4H-chromene* (3-TFC) and *2-amino-4-(4,5-methylenedioxyphenyl)-3-cyano-7-(dimethylamino)-4H-chromene* (4, 5-MC) were established with IR, ^1^H NMR, mass spectrometry and elemental analyses.

### Cell culture and morphological evaluation of the apoptotic cells

The HepG2, HCT116 and T47D cells were cultured in RPMI-1640 medium supplemented with 10 % fetal bovine serum (FBS), 2 mM L-glutamine, 50 IU/ml penicillin and 50 μg/ml streptomycin. For morphological studies, the cells were seeded in 12-well plates at 1 × 10^5^ cells/well and treated with indicated concentrations of the 3-BC, 3-NC, 3-TFC and 4, 5-MC for 72 h. Apoptosis was determined morphologically by Hoechst 33258 staining using fluorescence microscopy. In brief, cells were washed with cold phosphate-buffered saline (PBS) and adjusted to a density of 1 × 10^5^ cells per milliliter. Hoechst 33258 solution (1 mg/ml ddH_2_O) was added to the cell suspension at a final concentration of 100 μg/ml. Cellular morphology was evaluated by Axoscope two plus fluorescence microscopy (Zeiss, Germany).

### In vitro cytotoxicity assay

The HepG2, HCT116 and T47D cells (1 × 10^5^ cells/well) were cultured in 12 well cell culture plates for 24 h prior to treatment, then treated with indicated concentrations of the 3-BC, 3-NC, 3-TFC and 4,5-MC in a CO_2_ incubator for 72 h. At the end of this period, 10 μl of MTT (final concentration, 0.5 mg/ml) was added to each well and the plates were incubated for 4 h at 37 °C. Afterwards, 100 μl of the solubilization solution (0.04 N HCl in isopropanol) was added into each well and the absorbance values were determined at 570 nm using a microplate reader (Elx 800 Microplate Reader, Bio-TEK) [23].

### Annexin-V-FITC/PI double staining assays of the apoptotic cells

Double staining with FITC-Annexin V and PI for flow cytometry analyses was performed using phosphatidyle serine detection kit including FITC-Annexin V (IQ product, Netherland). The HepG2, HCT116 and T47D cells were cultured for 24 h and incubated with indicated concentrations (IC50 values) of 3-NC. The treated and/or untreated cells harvested after 72 h and washed twice with PBS, then resuspended in the binding buffer (calcium buffer, 100 μl). FITC-Annexin V (10 μl) was added to the cells followed by the addition of 10 μl PI. The samples were then incubated for 10 min in the dark at 4 °C and then subjected to flow cytometer (Becton Dickinson FACS) evaluation.

### Western blot analysis

HepG2, T47D and HCT116 cells were seeded and incubated for 24 h and then treated with indicated dose (IC50 values) of 3-NC. Following 48 h of treatment, the cells were harvested and lysed using loading dye containing, 1 % Triton X-100, 50 mM Tris-Cl (pH 6.8), 100 mM NaCl 1 % SDS, 10 % glycerol, β-mercaptoethanol and Bromophenol blue. Protein concentration of each sample was determined using Lowry’s method [24]. Equal quantities of protein (40-50 μg) were loaded on 12 % SDS-polyacrylamide gel electrophoresis (PAGE), and transferred to PVDF membranes. Transfer of proteins was assessed by ponceau-red staining. Non-specific binding membrane sites were blocked by incubation in the blocking buffer [Tris-buffered saline (TBS) buffer containing 0.1 % Tween 20 and 5 % non-fat dry milk] for 1 h at room temperature. Subsequently, the membranes were incubated with primary antibody (anti-β actin, anti-Bcl2 and anti-Bax) at 4 °C overnight. Thereafter, the membranes were washed in TBS, and incubated with HRP conjugated secondary antibodies for 1 h at room temperature. The proteins were detected using enhanced chemiluminescence (ECL) detection system (Thermo Scientific) [25]. Intensities of the bands were quantified using the NIH ImageJ software (http://rsb.info.nih.gov/ij/).

### Statistical analysis

Data were derived from at least three independent experiments and presented as means ± SD. Significant differences were determined using the unpaired Student’s *t*-test. Differences with p-values smaller than 0.05 were considered significant.

## Results

### Anti-proliferative activity of investigated 4-aryl-4H-chromene compounds

Growth inhibitory activity and IC50 values of 3-BC, 3-NC, 3-TFC and 4, 5-MC compounds were evaluated using MTT assay. HepG2, T47D and HCT116 cells, 1 × 10^5^ cells/ml were treated with various concentrations of the chromene compounds for 72 h. Cell viability was observed following the treatment of different doses of the compounds for 72 h. As shown in Table 1, cell viability was reduced following the treatments. Using these data, the IC50 values were obtained and summarized in Table 2. Cell viability of HepG2, T47D and HCT116 cells were reduced upon treatment with 3-BC, 3-TFC and 4, 5-MC compounds. The 3-BC, 3-TFC and 4, 5-MC inhibited the growth and viability of the cells with IC50 values of 60 ± 3.0, 80 ± 4.0 and 140 ± 3.0 nM in HepG2 cells, 65 ± 2.0, 75 ± 4.0 and 160 ± 5.0 nM in T47D cells and 50 ± 3.0, 75 ± 2.0 and 120 ± 4.0 nM in HCT116 cells, respectively (Table 2). Cell viability assays in HepG2, T47D and HCT116 cells treated with 3-NC were previously described [21] (Table 2). This compound was found to be highly active with IC50 values of 55 ± 2.0, 60 ± 3.0 and 50 ± 2.0 nM in HepG2, T47D and HCT116 cells, respectively [21]. As shown in Tables 1 and 2, the 3-NC was more active to the cells in comparison with other compounds. Furthermore, all cells were slightly less sensitive to compound 4, 5-MC. Among these compounds, 3-NC was selected for next studies.

**Table 1:** Cell viability in human cancer cells treated with various doses of the 4-aryl-4H-chromene derivatives for 72 h

				R		
						
Cell lines	Concentration (nM)	3-BC	3-NC^a^	3-TFC	Concentration (nM)	4, 5 MC
HepG2	0	100	100	100	0	100
	50	61 ± 2.5	57 ± 3.1	91 ± 3.1	100	67 ± 2.1
	55	53 ± 2.4	50 ± 4.2	89 ± 3.6	110	65 ± 1.6
	60	50 ± 3.2	33 ± 2.1	87 ± 2.5	120	46 ± 2.5
	65	43 ± 2.1	30 ± 3.6	82 ± 2.3	130	45 ± 2.7
	70	42 ± 3.1	28 ± 3.3	76 ± 3.2	140	42 ± 3.6
	75	40 ± 3.3	22 ± 1.2	65 ± 2.1	150	40 ± 2.5
	80	37 ± 1.8	20 ± 2.1	53 ± 3.7	160	28 ± 3.3
T47D	0	100	100	100	0	100
	50	80 ± 3.3	74 ± 1.5	86 ± 2.4	100	79 ± 3.5
	55	60 ± 2.5	64 ± 2.3	75 ± 5.3	110	68 ± 4.1
	60	53 ± 2.3	52 ± 3.5	72 ± 4.2	120	66 ± 4.2
	65	51 ± 3.1	48 ± 4.6	64 ± 4.4	130	58 ± 2.4
	70	48 ± 4.2	41 ± 2.6	61 ± 2.2	140	51 ± 3.2
	75	45 ± 2.1	36 ± 2.5	50 ± 4.2	150	46 ± 4.5
	80	42 ± 3.3	35 ± 1.3	44 ± 2.1	160	35 ± 3.2
HCT116	0	100	100	100	0	100
	50	50 ± 3.5	50 ± 3.1	85 ± 2.5	100	70 ± 3.5
	55	43 ± 2.4	42 ± 3.2	80 ± 3.6	110	67 ± 3.6
	60	35 ± 3.2	37 ± 2.5	73 ± 4.5	120	65 ± 2.5
	65	28 ± 2.1	30 ± 4.6	70 ± 3.3	130	62 ± 2.3
	70	22 ± 4.1	22 ± 3.3	70 ± 1.2	140	60 ± 3.2
	75	17 ± 3.1	15 ± 1.7	67 ± 3.4	150	54 ± 2.1
	80	12 ± 3.3	12 ± 2.8	65 ± 3.0	160	50 ± 3.7

Cell viability was evaluated by MTT assay. Data were expressed as a percentage of control measured in the absence of the compounds. Each point represents the mean ± SD of three independent experiments. (P < 0.05)

^a^Data from Ref. [21]

**Table 2:** Inhibition of cell proliferation by 4-aryl-4H-chromenes

		IC50^a^ (nM)	
Compound	HepG2	T47D	HCT116
3-BC	60 ± 3.0	65 ± 2.0	50 ± 3.0
3-NC	55 ± 2.0^b^	60 ± 3.0^b^	50 ± 2.0 ^b^
3-TFC	80 ± 4.0	75 ± 4.0	75 ± 2.0
4, 5 MC	140 ± 3.0	160 ± 5.0	120 ± 4.0

^a^Data are means of three or more experiments and are reported as means ± standard error of the mean (SEM)

^b^Data from Ref. [21]

### Morphological assay of the apoptotic cells

**Fig. 2:**
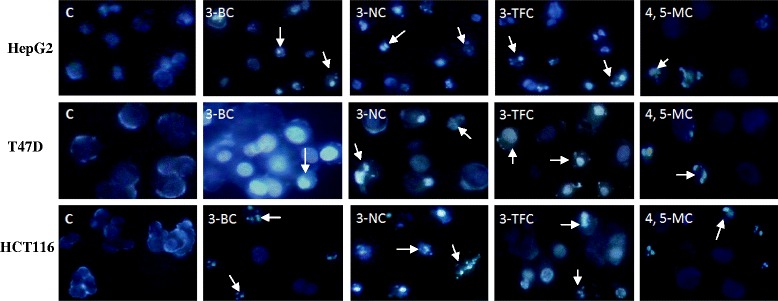
Fluorescence microscopy of the HepG2, T47D and HCT116 cells treated with the 3-BC, 3-NC, 3-TFC and 4, 5-MC (at IC50 values). Fluorescence images of the cells stained with Hoechst 33258 after 72 h. All of the four investigated chromenes induced condensation and fragmentation of the nuclei (arrows). Magnification, 200 ×

To induce apoptosis, cells were cultured at a density of 1 × 10^5^ cells/ml in the presence of indicated concentrations (IC50 Values) of the 3-BC, 3-NC, 3-TFC and 4, 5-MC for 72 h. We stained harvested cells with Hoechst 33258 to detect the apoptotic cells (Fig. 2). Under fluorescence microscope, the staining showed that the nucleus was large and round, without condensation and fragmentation in the control cells. However, cells treated with the compounds exhibited chromatin condensation and fragmentation, a typical morphological feature of apoptosis. These data indicate that the investigated compounds causes apoptosis in the three cell lines. Given high anti-proliferative activity of 3-NC compared to other chromene compounds (see Tables 1 and 2), the 3-NC was chosen for further studies.

### Apoptosis assay by flow cytometry

**Fig. 3:**
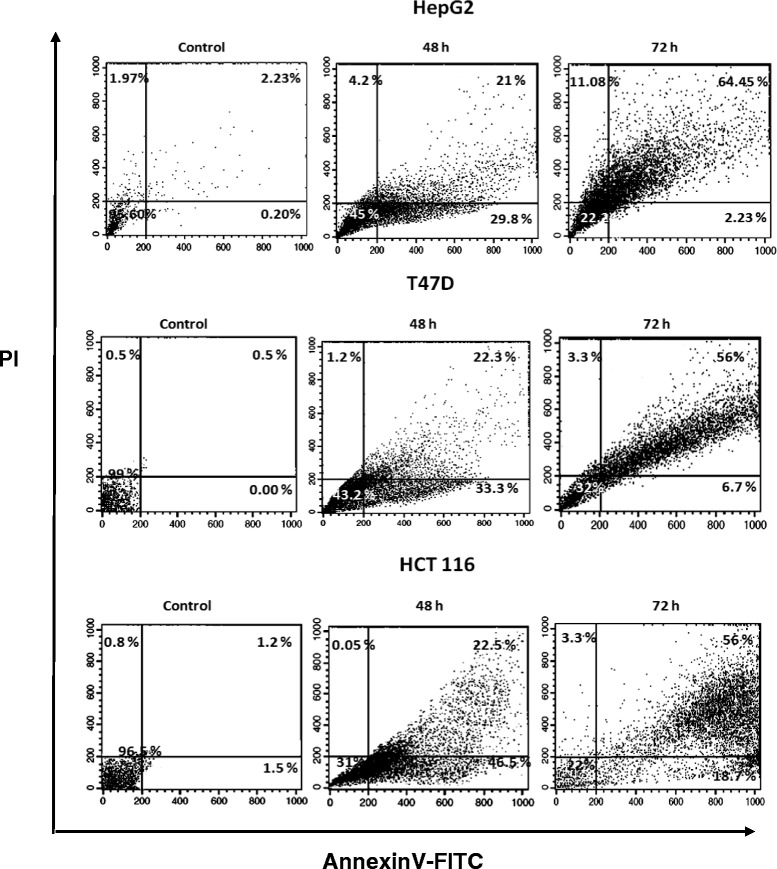
Flow cytometry apoptosis detection of the HepG2, T47D and HCT116 cells treated with the 3-NC. The cells were treated with indicated concentrations (IC50 values) of the 3-NC and harvested after 48 and 72 h for double staining (Annexin V/PI) flow cytometric assay. As is evident from Figure, after 72 h, a shift occurred from early apoptosis (lower-right quadrant) to late apoptosis or necrosis (upper-right quadrant). The results are those of two independent experiments

We used flow cytometric method to confirm apoptosis. The detection of surface exposed phosphatidyl-serine (PS) by AnnexinV-FITC has been shown to be a general and early marker of apoptosis as a result of redistribution of the plasma membrane of cells following the occurrence of apoptosis [26]. Consistent with previous data on morphological changing of cells, apoptosis was mostly observed after 72 h of exposure to the 3-NC (at IC50 values). Early apoptosis (lower right quadrant) and late apoptosis/necrosis (upper right quadrant) were clearly evident in dot plots of Fig. 3. Flow cytometric analysis using Annexin V/PI staining showed that treatment of the cells with the 3-NC causes more than 60 % of HepG2 and T47D and more than 70 % of HCT116 cells to die through apoptosis.

### Evaluation of Bcl2 and Bax proteins expression upon 3-NC treatment

**Fig. 4:**
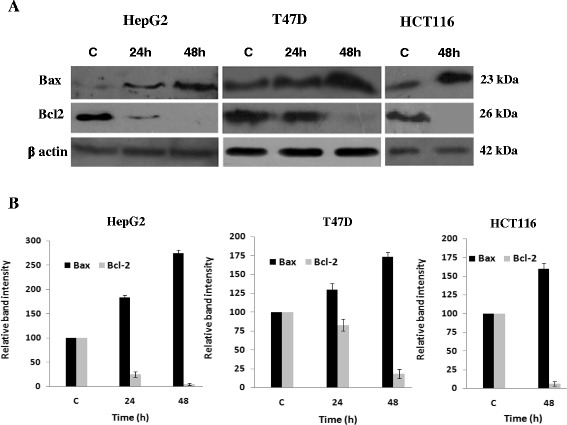
Expression level of Bcl2 and Bax proteins in the HepG2, T47D and HCT116 cells. **a** Following the treatment of the cells with indicated concentrations (IC50 values) and times (24-48 h) of the 3-NC, the cells were harvested and subjected to Western Blot analysis using polyclonal antibodies against Bcl2 and Bax. β-Actin was used as the loading control. **b** The protein levels of Bcl2 and Bax in control and treated cells were quantified by ImageJ software and normalized to β-actin band intensity. The relative intensities of the proteins over that of Actin for the control cells were set as 100 %. Each data was repeated three times (*n* = 3)

Recently, we reported the activation of caspase-9, caspase-3 and down regulation of IAP family members in HepG2, T47D and HCT 116 cells treated with the 3-NC [21]. Given that apoptosis inducing agents frequently signal through changes in the expression of Bcl2 family proteins, we now decided to examine possible alterations of Bcl2 and Bax proteins. Thus expression of Bcl2 (anti-apoptotic) and Bax (pro-apoptotic) before and after treatment with 3-NC at IC50 values was examined in these cell lines using western blot (Fig. 4a and b). Although Bcl2 expression in HepG2 cells was reduced dramatically after 24 h, its reduction in T47D was resistant to drug such that the major reduction occurred following 48 h of treatment. Following 48 h treatment, Bcl2 almost completely disappeared in all of the three cell lines. Simultaneous with down regulation of Bcl-2, expression of Bax was increased in all three cell lines upon treatment with 3-NC (Fig. 4). Bax expression in T47D and HCT116 cells increased by almost 50 %, while its expression was almost tripled in HepG2 cells indicating that HepG2 is the most sensitive cell to chromene treatment.

## Discussion

The induction of apoptosis is suggested to be an efficient strategy for treatment of cancer [27], because, cell numbers are dependent upon the extent of cell proliferation and death. However, cancer cells have developed various mechanisms to resist apoptotic cell death [27]. One of these mechanisms is over-expression of anti-apoptotic Bcl2 family proteins, which give rise to apoptosis resistance decreasing efficiency of therapeutics [28, 29]. The Bcl2 family of proteins is the central regulators of the mitochondrial cell-intrinsic apoptotic [30, 31]. The Bcl2 itself binds to pro apoptotic members such as Bax, preventing pore formation and cytochrome c release [31]. In contrast, increase in expression of Bax, induces cell death eliminating tumor cells [32, 33]. Given numerous reports underlining reduced expression of Bax and increased expression of Bcl2 in many drug-resistant tumor cells and recent reports showing the ability of 4-aryl-4H-chromenes family to induce procaspase-9 cleavage and thus activation we hypothesized that induction of mitochondrial apoptosis pathway by chromene compounds might be mediated through the Bcl2 and Bax proteins.

HepG2, T47D and HCT116 Cells were treated with various concentrations (IC50 values) of 3-NC, which caused strong induction of apoptosis in a time and dose dependent manner. These observations agree with the previous reports [21]. Interestingly, Bax and Bcl2 responded differently to the drug in a cell type dependent manner. For instance, levels of Bcl2 expression in HepG2 and HCT116 cells strongly decreased time-dependently after 24 and 48 h. In T47D cells high levels of Bcl2 were still seen after 24 h. However, prolonged exposure (48 h) to the drug, resulting in decreased Bcl2 expression levels (Fig. 3). The expression of Bax also changed in all of these cell lines after treatment with the drug in a time-dependent manner. In HepG2 and HCT116 cells, Bax level considerably increased following 48 h of treatment while in T47D cells, slightly increased after 24 and 48 h of treatment. The pattern of changes in the expression of Bax and Bcl2 is evidence for greater resistance to the drug in T47D cells and higher sensitivity in HCT116 cells (Figs. 2 and 3 and Table 1). Mechanism(s) by which 4-aryl-4H-chromenes modulate the Bcl2 and Bax protein levels remains unknown. Microtubule inhibitors as apoptosis-inducing agents [34]; observed mechanisms are phosphorylation of Bcl2 leading to protein degradation and induction of apoptosis [35]. These compounds (as microtubule inhibitors) might interfere with the expression of Bcl2 proteins or more likely activate degradation pathways of this protein [20]. However, it has been recently reported that ethyl 2-amino-6-bromo-4-(1-cyano-2-ethoxy-2-oxoethyl)- 4H-chromene-3-carboxylate (HA14-1) binds to Bcl2 protein, and blocks its anti-apoptotic function in HL-60 cells [15, 36]. Apoptotic effects of HA14-1 depend on Apaf-1 and activation of caspase-9, followed by caspase-3 [15]. It has been suggested that a high ratio of Bax to Bcl2 can lead to collapse of mitochondrial membrane potential, resulting in release of cytochrome c and consequently causes cell apoptosis [37, 38]. Our data also confirm that decreased Bcl2 protein expression, its inhibitory effect on Bax and caspase-9 was removed and leads to over expression of Bax and finally activation of caspase-9. Although, activation of caspase-9 also leads to loss of mitochondrial membrane potential by cleaving anti-apoptotic members of Bcl2 family including Bcl-xL and Bcl2 [39]. Therefore, altered ratio of pro-apoptotic and anti-apoptotic Bcl2 family members might be an important key question to understand the sensitizing effect of 3-NC in these cells. It has been greatly reported that Bcl2 is inactivated by phosphorylation when cells are arrested in G2/M [40, 41]. Furthermore, inhibition of tubulin polymerization and cell cycle arrest at G2/M phase has been reported for 4-aryl-4H-chromenes family [16–18]. Our data showed that the expression of Bcl2 proteins decreased after 24 and 48 h of 3-NC treatment. In contrast, considerable change was noted in the expression of Bax protein. Thus, it seems that the Bcl2 family of proteins might act as downstream signal in the process of 3-NC-induced apoptosis in these human cancer cells.

In previous studies, we have reported down-regulation of several IAPs such as XIAP, cIAP2 and survivin in HepG2, T47D and HCT116 human cancer cells [21]. The expression changes of apoptotic regulators such as Bcl2 and IAPs is an attractive strategy for exploring their role in raising the possibility of tumor cell apoptosis and thus defining potential therapeutic strategies. IAP proteins have been identified to inhibit apoptosis via their function as direct or indirect inhibitors of initiator as well as executioner caspases, regulating cell cycle progression and modulating receptor-mediated signal transduction [42, 43]. Our findings indicated that a cell death associated with the down-regulation of IAP proteins provides a powerful model for investigating mitochondrial control of cell death in these human cancer cells. These findings are compatible with the reports that over-expression of IAP family proteins inhibits apoptosis induced by Bax and other pro-apoptotic Bcl2 family proteins, which are known for their ability to target mitochondria and induce cytochrome c release [44]. Although, IAP proteins, do not interfere directly with Bax-mediated release of cytochrome c [45], some of the findings indicating that the human IAPs such as XIAP, c-IAP1, c-IAP2, and survivin block caspase activation and apoptosis downstream of Bax, Bak and cytochrome c [44, 45].

Then, these compound from 4-aryl-4H-chromenes family can be very effective in overcoming chemoresistance because they upregulate Bax and down regulate Bcl2 leading to cytochrome c release from one hand and antagonizing IAPs unleashing caspase-9 and 3 activities on the other hand. Combination of these effects on cell lines derived from various tissues may render chromenes as effective cancer therapy agents.

In conclusion, we have reported four compounds from 4-aryl-4H-chromenes family with high apoptotic activity. Induction of apoptosis by an active compound (3-NC) was achieved by down-regulation of Bcl2 and up-regulation of Bax. Our observations show that for an effective elimination of cancerous cells simultaneous down-regulation of Bcl2 following by over expression of Bax is a preferred approach and persistent expression of Bcl2 and IAP proteins can be a mechanism by which cancer cells exhibit resistance to chemotherapy.

## Abbreviations

3-NC: 2-amino-4-(3-nitrophenyl)-3-cyano-7-(dimethylamino)-4H-chromene
3-BC: 2-amino-4-(3-bromophenyl)-3-cyano-7-(dimethylamino)-4H-chromene
3-TFC: 2-amino-4-(3 trifluoromethylphenyl)-3-cyano-7-(dimethylamino)-4H-chromene
4: 5-MC, 2-amino-4-(4,5-methylenedioxyphenyl)-3-cyano-7-(dimethylamino)-4H-chromene
Apaf-1: Apoptotic factor-1
Bcl2: B-cell lymphoma 2
BH: Bcl homology
BIR: Baculoviral IAP repeat
Caspases: Cysteine-aspartic proteases
FBS: Fetal bovine serum
MTT: 3-(4, 5-dimethylthiazol-2-yl) 2, 5-diphenyl tetrazolium bromide
PBS: Phosphate-buffered saline
PS: Phosphatidyl-serine
IAPs: Inhibitor of apoptosis proteins
XIAP: X-Linked Inhibitor of Apoptosis Protein
cIAP: cellular inhibitor of apoptosis protein
